# Attenuation of Pathogenic Immune Responses during Infection with Human and Simian Immunodeficiency Virus (HIV/SIV) by the Tetracycline Derivative Minocycline

**DOI:** 10.1371/journal.pone.0094375

**Published:** 2014-04-14

**Authors:** Julia L. Drewes, Gregory L. Szeto, Elizabeth L. Engle, Zhaohao Liao, Gene M. Shearer, M. Christine Zink, David R. Graham

**Affiliations:** 1 Department of Molecular and Comparative Pathobiology, Johns Hopkins University School of Medicine, Baltimore, Maryland, United States of America; 2 Experimental Immunology Branch, Center for Cancer Research, National Cancer Institute, National Institutes of Health, Bethesda, Maryland, United States of America; 3 Johns Hopkins Bayview Proteomics Center, Department of Medicine, Division of Cardiology, Johns Hopkins School of Medicine Bayview Campus, Baltimore, Maryland, United States of America; Centro de Investigación en Medicina Aplicada (CIMA), Spain

## Abstract

HIV immune pathogenesis is postulated to involve two major mechanisms: 1) chronic innate immune responses that drive T cell activation and apoptosis and 2) induction of immune regulators that suppress T cell function and proliferation. Both arms are elevated chronically in lymphoid tissues of non-natural hosts, which ultimately develop AIDS. However, these mechanisms are not elevated chronically in natural hosts of SIV infection that avert immune pathogenesis despite similarly high viral loads. In this study we investigated whether minocycline could modulate these pathogenic antiviral responses in non-natural hosts of HIV and SIV. We found that minocycline attenuated *in vitro* induction of type I interferon (IFN) and the IFN-stimulated genes indoleamine 2,3-dioxygenase (IDO1) and TNF-related apoptosis inducing ligand (TRAIL) in human plasmacytoid dendritic cells and PBMCs exposed to aldrithiol-2 inactivated HIV or infectious influenza virus. Activation-induced TRAIL and expression of cytotoxic T-lymphocyte antigen 4 (CTLA-4) in isolated CD4+ T cells were also reduced by minocycline. Translation of these *in vitro* findings to *in vivo* effects, however, were mixed as minocycline significantly reduced markers of activation and activation-induced cell death (CD25, Fas, caspase-3) but did not affect expression of IFNβ or the IFN-stimulated genes IDO1, FasL, or Mx in the spleens of chronically SIV-infected pigtailed macaques. TRAIL expression, reflecting the mixed effects of minocycline on activation and type I IFN stimuli, was reduced by half, but this change was not significant. These results show that minocycline administered after infection may protect against aspects of activation-induced cell death during HIV/SIV immune disease, but that *in vitro* effects of minocycline on type I IFN responses are not recapitulated in a rapid progressor model *in vivo*.

## Introduction

In the absence of antiretroviral therapy, the majority of HIV-infected patients develop chronic immune activation, immunosuppression and eventually AIDS in a disease process representative of HIV/SIV infection in non-natural hosts (NNH). In contrast, most natural hosts (NH) of SIV such as African green monkeys, gorillas, and sooty mangabeys avert immune pathogenesis despite high viral loads [1]–[4]. One hypothesis for the different outcomes in NH versus NNH focuses on host immune responses to the virus. Documented immune differences include differential induction of type I IFN and IFN-stimulated genes (ISGs) such as TNF-related apoptosis inducing ligand (TRAIL), as well as the immune regulators cytotoxic T-lymphocyte antigen-4 (CTLA-4) and indoleamine 2,3-dioxygenase (IDO), all of which contribute to T cell dysfunction and potentially the development of AIDS [5]–[12]. IDO activity and TRAIL (both soluble and membrane-associated) remain elevated even in patients on suppressive cART [13]–[15]. In addition to their purported role in chronic HIV/SIV infection, many of these genes are also thought to be involved in the “cytokine storm” that contributes to pervasive inflammation, lymphocytopenia, and apoptosis during highly pathogenic influenza infections [16]–[19]. These immune responses therefore represent potential therapeutic targets for not only HIV infection but also other pathogenic viral infections such as influenza.

In the first arm of HIV pathogenesis, hyperactive innate immune responses that are driven by viral stimulation of Toll-like receptors (TLRs) and other pattern recognition receptors lead to chronic ISG expression, cellular activation, and apoptosis. Type I IFN responses are critical in the control of many viral infections, as ISGs can directly inhibit viral replication [20] and also stimulate adaptive immune responses [21]. Although type I IFN can inhibit HIV replication *in vitro*, administration of exogenous type I IFN has only a moderate effect on HIV viral load and disease progression *in vivo* [22]–[26]. Studies comparing NH and NNH have corroborated the hypothesis that hyperactive innate immune responses contribute to pathogenesis, as both NH and NNH express robust levels of ISGs during acute infection *in vivo* [5]–[7], but NH control this response within weeks while levels of IFNα mRNA and ISGs remain elevated in NNH throughout chronic infection [5]–[9]. These ISGs include TRAIL and Fas ligand (FasL), which can induce apoptosis of uninfected CD4+ T cells expressing the cognate receptors death receptor 5 (DR5) and Fas, and programmed death ligand 1 (PDL1), which can induce apoptosis or exhaustion of CD8+ T cells expressing the corresponding receptor programmed cell death 1 (PD1) [27]–[30]. Thus, despite the need for an early phase of IFN expression to stimulate adaptive immune responses and assist in the control of virus replication, chronic expression of type I IFN and ISGs likely does more harm than good in HIV/SIV infection of NNH [31].

In the second arm of HIV pathogenesis, induction of the immune regulators CTLA-4 and IDO suppresses the ability of T cells to proliferate and respond to antigen, further compromising immune responses already damaged by chronic ISGs. CTLA-4 is expressed on activated CD4+ T cells and CD4+Foxp3+ regulatory T cells (Tregs). These CTLA-4-expressing T cells convert DCs into regulatory DCs by ligating with B7 molecules and inducing IDO expression [32], [33]. IDO can also be upregulated in plasmacytoid DCs (pDCs) by stimulation with pro-inflammatory cytokines such as IFNα, β, and γ, TNFα, or directly by HIV [34]. Alternatively, long-term, chronic expression of IDO in regulatory pDCs may be mediated by the anti-inflammatory cytokine TGFβ [35], [36]. IDO-expressing pDCs mediate suppressive effects on T cells via 1) degradation/depletion of local tryptophan which prevents T cell proliferation and 2) generation of kynurenine and downstream metabolites that block T cell proliferation [37], induce T cell apoptosis [38], [39], and convert naïve CD4+ cells and Th17 cells into Tregs [40], [41]. Thus, CTLA-4 and IDO work in tandem to increase the number and regulatory function of suppressive DCs and Tregs in lymphoid tissues. Notably, NNH with progressive infections have higher levels of CTLA-4, Foxp3, and IDO mRNA in their lymphoid tissues compared to non-progressors and uninfected individuals [10]–[12]. These data suggest that CTLA-4+Foxp3+ Tregs accumulate in lymphoid tissues during progressive infection of NNH, where they can influence the function of DCs and other T cells, and thereby represent an important therapeutic target in preventing immune suppression in HIV [42].

Minocycline, a semi-synthetic tetracycline derivative, ameliorates the severity of a number of inflammatory diseases, including rheumatoid arthritis [43] and animal models of multiple sclerosis [44], amyotrophic lateral sclerosis [45], Huntington’s disease [46], Parkinson’s disease [47], allergy/asthma [48], [49], Japanese encephalitis virus [50], and SIV-associated neurological disease [51], [52]. Minocycline’s effects have been primarily attributed to its ability to decrease activation of a variety of immune cell types, including monocytes/macrophages, microglia, and T cells [53]–[56]. Additionally, many inflammatory cytokines can be downregulated by minocycline, including IL-6 [52], [57]–[59], IL-1β [58], TNFα [57]–[60], and IFNγ [59]–[61]. However, minocycline’s effects on type I IFN responses and IDO have not been explored.

Here we show that minocycline prevented the pathogenic upregulation of type I IFN and IDO in pDCs following *in vitro* exposure to aldrithiol-2-inactivated (AT-2) HIV and prevented activation of CD4+ T cells after exposure to anti-CD3 and type I IFN, culminating in decreased surface TRAIL expression on both cell types. The AT-2 inactivated form of HIV was used in order to dissociate innate immune signaling from factors associated with productive infection because pDCs can be infected by HIV. Reductions in type I IFN and TRAIL with minocycline treatment were also observed in PBMCs exposed to either AT-2 HIV or infectious influenza virus. A trend towards reduced TRAIL expression was also seen in spleens from minocycline treated, SIV-infected pigtailed macaques and was accompanied by downregulation of the cellular activation marker CD25 and the apoptosis-promoting genes Fas and caspase-3. Notably, these changes in CD25, Fas, and caspase-3 upon minocycline treatment were not significantly different from the changes seen in animals treated with combination antiretroviral therapy (cART). However, minocycline did not reduce expression of ISGs in the spleens of SIV-infected macaques. Taken together, our data suggest that minocycline is capable of attenuating aspects of pathogenic immune responses during HIV/SIV infection and may be useful as an adjunct immunotherapy against hyperactive immune responses in diverse viral infections.

## Materials and Methods

### Ethics Statement on Human Subjects

For *in vitro* experiments, blood was drawn from healthy human donors following their informed oral consent in accordance with the Johns Hopkins Medicine Institutional Review Board protocol NA 00014329 or via anonymous donor leukopaks from the Johns Hopkins University Outpatient Center or New York Blood Bank. The Johns Hopkins Medicine Institutional Review Board approved all experiments involving healthy human donors and approved the use of informed oral consent in place of written consent.

### Ethics Statement on Animal Subjects

All animal studies were approved by the Johns Hopkins Animal Care and Use Committee (IACUC protocol #PR12M310); all animals were humanely treated in accordance with federal guidelines and institutional policies. All juvenile/adult pigtailed macaques (*Macaca nemestrina*) used in this study were obtained from nonhuman primate breeding facilities within the United States. A total of thirty-four pigtailed macaques were studied. Median age was 3 years (1.4–9.5 years); median weight was 3.5 kg (2.4–14.5 kg). Of the 34 animals, 7 were used as uninfected controls and 27 were inoculated intravenously with SIV/DeltaB670 and SIV/17E-Fr, as previously described [62]. All animals were male with the exception of 2 procedural controls that were female. Eleven of the SIV-infected macaques received no drug treatment. Eleven of the SIV-infected macaques received minocycline at a dose of 4 mg/kg per day divided over two doses and administered orally starting at 21 days p.i. as previously described [51]. No adverse effects due to minocycline treatment were observed in these animals. Five of the SIV-infected macaques received combination antiretroviral therapy (cART) beginning at day 12 p.i. which suppressed plasma viremia to <100 SIV RNA copy eq./mL by day 70 p.i. [63]. The 4-drug cART regimen consisted of 270 mg/kg of the protease inhibitor atazanavir (Bristol-Myers Squibb), 10 mg/kg of the integrase inhibitor L-870812 (Merck), and 205 mg/kg of the protease inhibitor saquinavir (Roche) administered orally twice/day; as well as 30 mg/kg of the nucleotide reverse transcriptase inhibitor PMPA (Gilead) administered subcutaneously once daily.

Macaques were housed in Johns Hopkins University facilities which are fully accredited by the association for the Assessment and Accreditation of Laboratory Animal Care, International, (AALAC). Macaques were housed in pairs or groups prior to inoculation, then housed singly after inoculation to prevent repeated infection events, in stainless steel cages providing at least 6 square feet of space per animal and with visual and auditory contact with conspecifics. All housing met or exceeded guidelines of the National Institutes of Health “Guide for the Care and Use of Laboratory Animals” and the United States Department of Agriculture Animal Welfare Act. Macaques were fed a balanced, commercial macaque chow (Purina Mills, Gray Summit, MO, USA) once a day supplemented with a variety of food enrichment. Water was provided *ad libitum* and a light:dark cycle of 14∶10 hours was maintained. Macaques were provided with environmental enrichment including manipulanda, novel foodstuffs and movies/radio under the supervision of an enrichment specialist. Macaques were observed 1–2 times daily by trained laboratory and/or veterinary staff, and attitude, appetite and fecal consistency noted; macaques were weighed and plasma and CSF viral loads monitored at least once every two weeks. If abnormalities were noted, veterinary personnel were consulted and treatment initiated if necessary. Euthanasia was performed under veterinary supervision using an overdose of intravenous sodium pentobarbital while under deep ketamine sedation (10 mg/kg intramuscular), followed by perfusion with 1X PBS prior to tissue harvest. All infected, untreated macaques were euthanized during late-stage infection at approximately 84 days p.i., a timepoint at which the majority of infected animals develop encephalitis [64], or prior to this timepoint if macaques presented with clinical symptoms as previously described [65]. Minocycline-treated animals were also euthanized at approximately 84 days p.i., while cART-treated animals were euthanized at approximately 180 days p.i., after approximately 100 days of plasma virus suppression. ARRIVE guidelines for animal research are reported in Checklist S1.

### Minocycline Dosage *In vitro*

A dose of 20 μM minocycline was chosen based on *in vitro* dose-response testing on pDC viability as well as a review of pharmacokinetic studies of patients given a commonly prescribed dose of minocycline (oral 200 mg/day), where plasma concentrations of minocycline reached 3.0–3.6 mg/L, roughly equivalent to 6–7 μM [66]. Since minocycline is absorbed into tissues at a high rate (up to 10 times the amount in plasma) [67], an *in vitro* dose of 20 μM minocycline is a physiologically relevant concentration that could be feasibly obtained in tissue microenvironments.

### PBMC Isolation

Whole blood from healthy human donors was obtained in accordance with the Johns Hopkins IRB protocol NA 00014329 or via leukopaks from the Johns Hopkins University Outpatient Center or New York Blood Bank. PBMCs were isolated from whole blood via Ficoll-Hypaque density gradient centrifugation and red blood cell lysis (10 minute incubation at 37°C with 1X solution of the following 10X buffer: 4.15 g NH_4_Cl, 0.5 g KHCO_3_, 0.15 g EDTA dissolved in water and adjusted to pH 7.2–7.3 in a final volume of 500 mL). PBMCs were subsequently washed with HBSS with EDTA before further use.

### pDC Isolation and Culture

pDCs were isolated from healthy human PBMCs via the Diamond pDC Isolation Kit (Miltenyi). Purity was consistently >95% as determined by BDCA-2 and CD123 flow cytometry staining. pDCs were plated at a density of 50,000 cells/well in flat-bottom 96-well plates in 150 μL RPMI 1640 supplemented with 10% FBS, 2 mM L-glutamine, 1 mM HEPES buffer, and 1% Pen-Strep (Invitrogen; final concentration 100 U/mL penicillin and 100 μg/mL streptomycin). Ten ng/mL IL-3 (R&D Systems) was added to the media daily. pDCs were co-treated with 20 μM minocycline hydrochloride (Sigma) dissolved in warm media and 300 ng/mL p24 equivalents of AT-2 HIV-1_MN_ (X4-tropic, strain P3935, AIDS and Cancer Vaccine Program, SAIC-Frederick, a gift of Dr. Jeffery Lifson and Julian Bess). After 18 hours, cell-free supernatants were collected and stored at −80°C and cells were either analyzed by flow cytometry or lysed for RNA extraction.

### CD4+ T Cell Isolation and Culture

CD4+ T cells were isolated from PBMCs by the Dynabeads FlowComp Human CD4 kit (Invitrogen). Purity was consistently >95% of live cells as determined by CD3 and CD4 staining by flow cytometry. CD4+ T cells were grown in 96-well plates or anti-CD3 coated 96-well plates (BD) with or without 20 μM minocycline hydrochloride (Sigma) in 100 μl RPMI 1640 supplemented with 10% FBS, 2 mM L-glutamine, 1 mM HEPES buffer, and 2 mg/mL gentamicin. After 24 hours, a 50 μL aliquot containing 60 μM minocycline was added to replenish minocycline at a final concentration of 20 μM in the 150 μL well volume. Some wells were also stimulated with 1,000 U/mL each of IFNα-2a and IFNβ-1a (PBL). All wells had a final volume of 150 μL and were cultured for an additional 24 hours (total 48 hours) before flow cytometry analysis.

### PBMC Treatment with Virus and Minocycline

PBMCs were seeded in 24-well plates at a density of 4–5 million cells/mL in RPMI 1640 supplemented with 10% FBS, 2 mM L-glutamine, 1 mM HEPES buffer, and 10 U/mL recombinant IL-2 (BD Biosciences). Cells were treated with varying doses of minocycline hydrochloride (Sigma; 0, 20, or 40 μM) dissolved in warm media for 2 hours before adding varying doses of either AT-2 HIV-1_MN_ or infectious influenza virus (A/Hong Kong/68-X-31 (H3N2)). AT-2 HIV concentrations are shown as ng/mL p24 equivalents while influenza concentrations are shown as ng/mL nucleoprotein, as measured by the Influenza A Nucleoprotein Antigen Capture ELISA (Virusys). After overnight culture, cells were harvested for flow cytometry.

### Flow Cytometry

Cells were removed from plates with gentle pipetting, washed with 1 x PBS, and stained for 1 hour at room temperature in the dark. PBMCs were stained with TRAIL antibody and gated on lymphocytes by forward and side scatter profiles. Isolated pDCs were stained with Annexin V, TRAIL, and 7AAD antibodies to discriminate viable cells. Isolated CD4+ T cells were stained with CD3 and TRAIL antibodies and gated on the lymphocyte morphology gate as determined by forward and side scatter. All antibodies were from BD. Samples were washed with 1 x PBS to remove excess antibodies and run on a BD FACSCalibur machine (pDC, CD4+ T cell experiments) with appropriate isotype controls or on a BD LSRFortessa (PBMC experiments). Data were analyzed by FlowJo software.

### IFN ELISAs

Cell-free supernatants from pDCs were analyzed by ELISA for IFNα (PBL Interferon Source #41100, which primarily recognizes 7/16 subtypes) and IFNβ (Invitrogen, with an extended initial incubation of 18 hours shaking at 4°C instead of 1 hour). Cell-free supernatants from PBMCs were analyzed by ELISA for IFNα (PBL Interferon Source) and IFNβ (PBL Interferon Source). All plates were read on a microplate reader (BioRad) at the recommended wavelengths for each respective ELISA assay.

### RNA Extraction from pDCs

RNA from human pDCs was isolated using the RNeasy Plus kit (Qiagen) followed by treatment with RQ1 DNase (Promega) for 30 min at 37°C and inactivation with Stop Solution (Promega) for 10 min at 65°C.

### RNA Extraction from Macaque Spleen

RNA was harvested from 25 mg of snap-frozen macaque spleen tissue by RNA STAT-60 (Isotex Diagnostics) extraction, DNase treatment with RQ1 DNase (Promega) for 1 hour at 37°C, and finally purification with miRVana Kit (Invitrogen). RNA concentration was determined by NanoDrop (ThermoFisher) and subsequently diluted to 40 ng/μL for Nanostring nCounter gene expression analysis. All samples underwent RNA integrity testing, and samples with RNA integrity scores less than 5 were excluded.

### qRT-PCR

RNA was reverse transcribed into cDNA with SuperScript III (Invitrogen). PCR amplification of cDNA was performed in a Chromo4 or CFX96 thermal cycler (both from BioRad) with Multiplex NoRox PCR Mix (Qiagen) and gene-specific primers and probes. Results were analyzed via the ΔΔCt method [68] with normalization to both 18S rRNA and negative controls. The following primers and probes were used for the *in vitro* human samples: IDO1 (5′- TGCTTTGACGTCCTGCTGG, 5′- TTCCTGTGAGCTGGTGGCA, and 5′- TXR - ATGCTGCTCAGTTCCTCCAGGACA - IAbRQs); Mx (5′- AGGAGTTGCCCTTCCCAGA, 5′- TCGTTCACAAGTTTCTTCAGTTTCA, and 5′- Hex - ACCAGCGGGCATCTGGTCACGA - BHQ1); 18S rRNA (5′- TAGAGGGACAAGTGGCGTTC, 5′- CGCTGAGCCAGTCAGTGT, and 5′- Cy5 - AGCAATAACAGGTCTGTGATG - BHQ2 or Crimson); IFNβ (5′- GCCTCAAGGACAGGATGAACTT, 5′- GCGTCCTCCTTCTGGAACTG, and 5′- Cy5 - CATCCCTGAGGAAATTAAGCAGCCGC - BHQ2). For some *in vitro* human samples, negative controls for IFNβ and IDO1 mRNA were not detectable and were assigned a Ct of 45 (limit of detection). The above sequences for IFNβ, Mx, and 18S as well as identical thermal cycling conditions were used for analysis of pigtailed macaque spleen RNA. Because IFNβ is an intronless gene, RNA samples underwent an additional DNase step with Turbo DNase (Invitrogen Life Technologies) for 30 min at 37°C to eliminate any residual genomic DNA immediately prior to IFNβ testing. A subset of the macaque samples were normalized in a second reaction to the lymphocyte gene CD2 (Invitrogen Life Technologies TaqMan Assay Rh02839718_m1), which was one of the most stable transcripts in the Nanostring nCounter assay across the samples used in this study. Normalization to CD2 yielded similar results to those from 18S normalization.

### Nanostring nCounter Gene Expression Analysis

Nanostring analysis allows for the highly sensitive and reproducible detection of mRNA molecules without the need for enzymatic amplification [69]. As part of a larger collaborative project, a CodeSet for 116 macaque genes (including the 7 transcripts analyzed in this manuscript as well as 11 putative housekeeping genes) for 96 samples were designed according to NanoString specifications based on rhesus macaque (*Macaca mulata*) and human sequences. Two hundred ng of each RNA sample were hybridized for 16 hours with the CodeSet, and genes were quantitated using the nCounter Digital Analyzer. Thirty-four of the 96 samples were examined in this study; the rest were excluded from the present analysis. Data were first normalized by the geometric mean of six spiked positive controls to correct for assay efficiency. Background was assessed as the average of eight spiked negative controls plus two standard deviations following positive control correction. Any genes with values below this threshold of 30 counts were excluded from the present analysis, with the exception of SIV/17E-Fr for which below threshold values were expected and obtained for uninfected and cART-treated animals. As no single housekeeping gene has been proven to be consistently expressed across different cell types, cell maturation states, tissue types, or disease models [70]–[74], we used the Kruskal-Wallis non-parametric analysis of variance (ANOVA) test on all positive control normalized genes to find the three most stably expressed genes across our spleen samples [70], [75]. We found that the lymphocyte markers CD7, CD2, and CD5 were the least varying genes between infection groups (*p* values = 0.953, 0.937, 0.828, respectively). The geometric mean of these three genes was used to normalize the positive control corrected data [74]. Alternative normalization to lymphocyte genes such as CD4 in HIV/SIV infected lymphoid tissue has been used in other studies as well [10], [11], [76]. Similar results were obtained when our data was normalized to the most stably expressed housekeeping gene, ribosomal gene RPS9 (Fig. S1). All other traditional housekeeping genes in our panel (ACTB, B2M, GAPDH, HPRT1, HMBS, RPL13A, SDHA, TBP, UBC, and YWHAZ) had Kruskal-Wallis *p*-values<0.100. Raw data as well as positive and negative controls are shown in Table S1. Trends in SIV viral load and IDO1 mRNA expression in spleen have been independently verified for subsets of the animals by qRT-PCR and kynurenine/tryptophan metabolite ratios, respectively (data not shown).

### Statistics

TRAIL and CTLA-4 in isolated pDCs and CD4+ T cells passed the Kolmogorov-Smirnov test for Gaussian distribution and were analyzed by paired *t*-test. ELISAs were analyzed by Wilcoxon paired *t*-test for nonparametric data. qRT-PCR data on *in vitro* experiments were analyzed by Wilcoxon paired *t*-test to compare different treatments. PBMC data on dose-response curves of minocycline with either AT-2 HIV or influenza were analyzed by two-way repeated measures ANOVA. *In vivo* spleen RNA data were analyzed by the Mann-Whitney test for nonparametric, unpaired data. All tests were performed with Prism software (version 5 or 6).

## Results

### Minocycline Attenuated IFN Responses in pDCs

**Figure 1:**
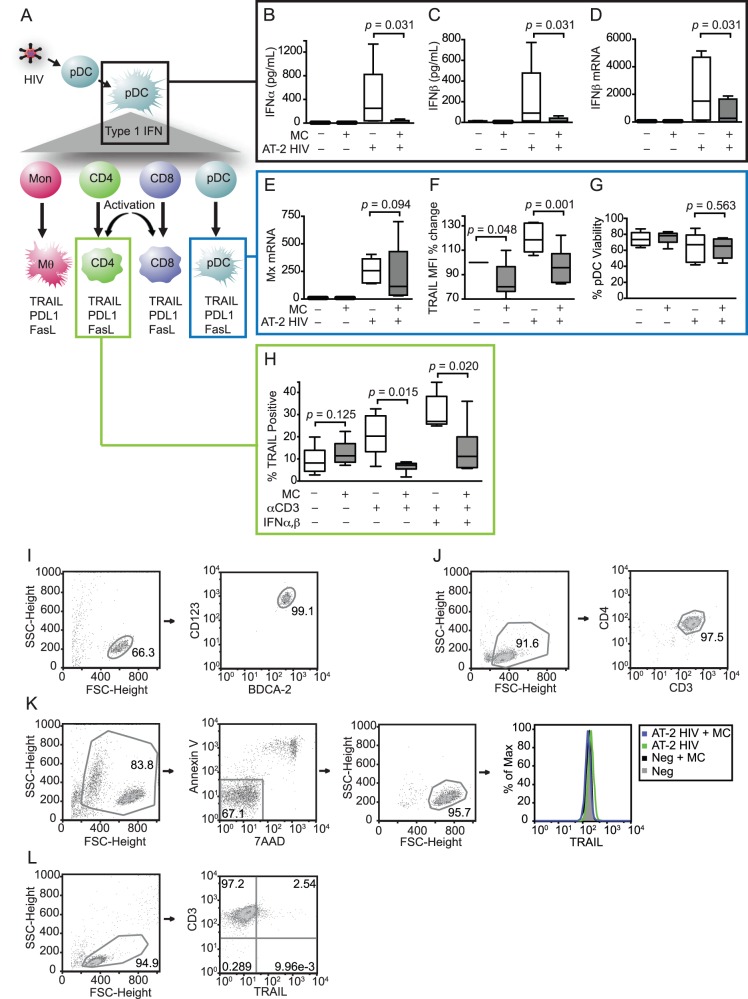
Minocycline prevents TRAIL upregulation in pDCs and CD4+ T cells by attenuating anti-viral IFN and activation responses. (A) pDCs become activated in response to TLR7/9 stimulation by HIV and secrete type I IFN, which in turn upregulates ISGs on leukocytes, including the TNF family members TRAIL and FasL and the B7 family member PDL1. These ligands induce apoptosis and/or exhaustion on target cells expressing the cognate death receptors (TRAIL/DR5; FasL/Fas; PDL1/PD1). (B–G) pDCs were isolated from blood of healthy human donors and exposed to 300 ng p24 eq./mL of AT-2 HIV with or without 20 μM minocycline for 18 hours (*n* = 6 different donors). (B, C) IFNα and IFNβ protein from pDC supernatants were measured by ELISA. (D) IFNβ mRNA was measured by qRT-PCR. (E) Mx mRNA and (F) TRAIL were measured as examples of ISGs by qRT-PCR and flow cytometry, respectively. (G) Viability of pDCs was determined by Annexin V/7AAD staining. (H) CD4+ T cells were isolated from blood of healthy human donors and activated with anti-CD3 with or without 20 μM minocycline (*n* = 6 different donors). After 24 hours, minocycline was replenished and some wells were additionally stimulated with IFNα and IFNβ. TRAIL was measured by flow cytometry after an additional 24 hours. (I) Representative flow cytometry gating of pDC purity by BDCA2+/CD123+ double staining immediately following isolation. (J) Representative gating of CD4+ T cell purity by CD4+/CD3+ double staining immediately following isolation. (K) Representative gating of pDC viability (Annexin V-/7AAD-) and TRAIL expression after 18 hours of stimulation in culture with virus and/or minocycline. (L) Representative gating of TRAIL expression in CD4+ T cells following 48 hours in culture. Parametric data were analyzed by paired *t*-test (TRAIL flow cytometry), and nonparametric data were analyzed by Wilcoxon signed-rank test (IFN ELISAs and IFNβ, Mx qRT-PCR).

As diagrammed in Fig. 1A, the elevated expression of type I IFNs and downstream ISGs such as TRAIL, FasL, and PDL1 in chronic infection of NNH compared to NH suggests that IFN responses are a point of divergence between pathogenic and nonpathogenic HIV/SIV infection and represent a potential target for immunotherapy [5]–[9]. To test whether minocycline could modulate these hyperactive innate immune responses, we first exposed pDCs to AT-2-inactivated HIV in the presence or absence of 20 μM minocycline. pDCs are the body’s professional type I IFN-producing cells, and although their number and function in chronic HIV/SIV infection are controversial [77]–[80], during acute HIV/SIV infection pDCs are known to be recruited to lymphoid tissues where they contribute to the innate immune response [81]–[83]. Because pDCs can be productively infected with HIV, we used AT-2 HIV to separate innate immune signaling from factors associated with productive infection [84].

Minocycline treatment prevented secretion of both IFNα (Fig. 1B; *p* = 0.031) and IFNβ (Fig. 1C; *p* = 0.031, respectively) by pDCs in response to AT-2 HIV stimulation, maintaining type I IFNs in the supernatant at nearly undetectable levels. We also examined IFNβ mRNA levels and found that minocycline-treated, AT-2 HIV-exposed cells exhibited significantly lower levels of IFNβ mRNA than cells exposed to virus alone (Fig. 1D; *p* = 0.031), indicating at least a partial block of the type I IFN response at the transcriptional level.

We also measured the expression of the ISGs myxovirus resistance A (Mx) and TRAIL in virus-exposed pDCs. Minocycline treatment reduced the median Mx mRNA expression to approximately half that induced by virus alone. However, this decrease in Mx was not significant (Fig. 1E; *p* = 0.094) despite reductions in 5 of 6 donors. Although there was incomplete suppression of Mx by minocycline, the ISG TRAIL was significantly downregulated by minocycline even in control samples without virus stimulation (Fig. 1F; *p* = 0.048). Minocycline also suppressed TRAIL (Fig. 1F; *p* = 0.001) to control levels in samples exposed to AT-2 HIV. The dosage of minocycline used in these studies (20 μM) did not affect pDC viability (Fig. 1G; *p* = 0.563, virus alone compared to virus + MC). These data confirm that minocycline inhibits IFN responses in isolated pDCs, decreasing their expression of TRAIL, which potentially inhibits their ability to cause TRAIL-mediated bystander T cell apoptosis.

### Minocycline Prevented Activation-induced TRAIL Expression on CD4+ T Cells

Because TRAIL upregulation in T cells can be mediated by type I IFN and/or activation through the TCR [85], we stimulated isolated CD4+ T cells from healthy human donors with anti-CD3 alone or in combination with type I IFNs (IFNα-2a and IFNβ-1a) in the presence or absence of minocycline. TRAIL was upregulated on CD4+ T cells after 48 hours of activation with anti-CD3 alone (Fig. 1H; *p* = 0.054) and with anti-CD3 plus IFNα and β (*p* = 0.002) when compared to unstimulated controls. Minocycline prevented upregulation of TRAIL in response to both anti-CD3 alone and anti-CD3 with exogenous IFN (*p* = 0.015, *p* = 0.020, respectively). These data suggest that minocycline can attenuate TRAIL upregulation *in vitro* via both inhibition of IFN signaling in pDCs and inhibition of activation signals through the TCR in CD4+ T cells.

### Representative Flow Cytometry Gating of Isolated pDCs and CD4+ T Cells

Representative flow cytometry gating for the purity of isolated pDCs and CD4+ T cells is shown in Fig. 1I–J. The purity of isolated pDCs (Fig. 1I) was tested by first gating on general cells (which excludes debris) by forward scatter (FSC) and side scatter (SSC), then by dual labeling for CD123 and BDCA-2 according to the manufacturer’s instructions to specifically identify pDCs from blood cell populations. The purity of isolated CD4+ T cells (Fig. 1J) was tested by using FSC and SSC gating for general cells (excluding cell debris), then for dual labeling with CD3 and CD4.

Representative flow cytometry gating on pDCs and CD4+ T cells following cell culture are shown in Fig. 1K–L. After 18 hours in culture, viability of the pDCs was examined by exclusion staining for Annexin V and 7AAD, markers for apoptosing and dead cells, respectively (Fig. 1K). TRAIL expression was then examined on the viable cell population (Annexin V-/7AAD-). A representative histogram of TRAIL expression in negative controls and AT-2 HIV stimulated cells with or without minocycline is also shown. pDC expression of TRAIL was high for all conditions examined; as a result, TRAIL data in Fig. 1F was expressed as a percent change rather than percent positive. TRAIL expression on isolated CD4+ T cells following culture for 48 hours was analyzed by gating on lymphocyte morphology by FSC and SSC, then by the percentage of CD3+ T cells expressing TRAIL (Fig. 1L).

### Minocycline Attenuated HIV- and Influenza-mediated Type I IFN Responses in PBMCs

**Figure 2:**
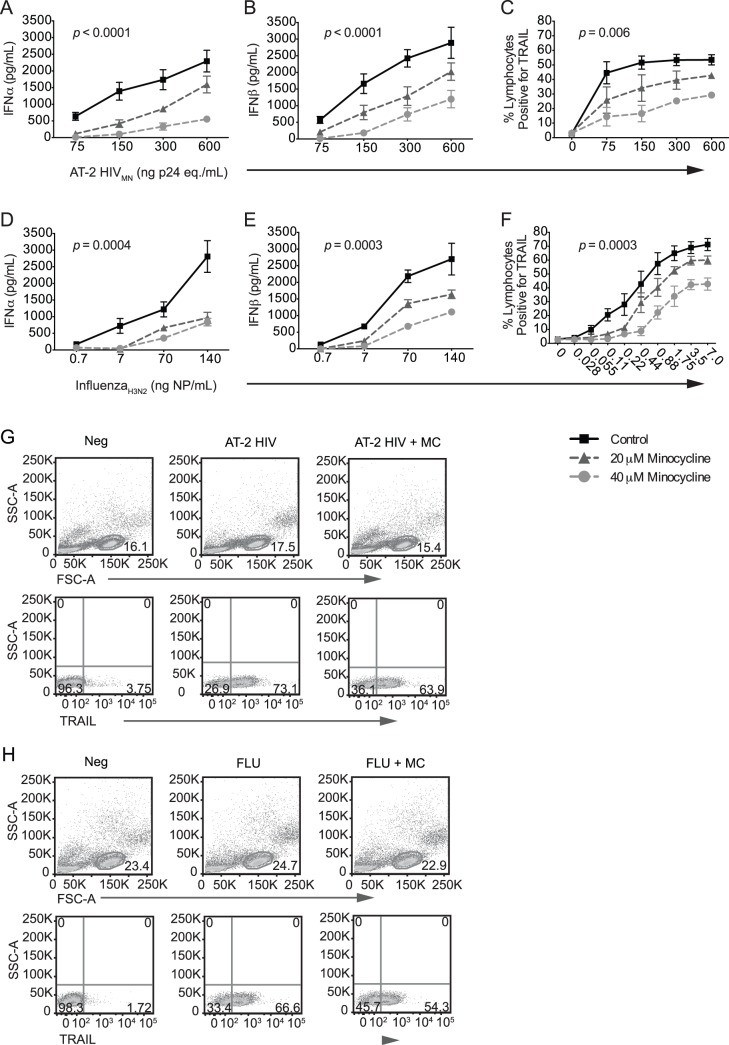
Minocycline attenuates type I IFN production and TRAIL expression in lymphocytes. PBMCs were isolated from the blood of healthy human donors, pretreated for two hours *in vitro* with 0, 20, or 40 μM minocycline, and exposed to increasing amounts of either AT-2 inactivated HIV (*n* = 4 different donors) or infectious influenza virus (*n* = 3 different donors). After overnight culture, supernatants were analyzed for secreted IFNα (**A, D**) and IFNβ protein (**B, E**) by ELISA. (**C, F**) Lymphocytes were analyzed by flow cytometry for TRAIL expression. (**G**) Representative flow cytometry gating of lymphocyte TRAIL expression in PBMC mixed cultures following AT-2 HIV stimulation. (**H**) Representative gating of lymphocyte TRAIL expression in PBMC mixed cultures following influenza stimulation. A two-way repeated measures ANOVA was used to compare the effect of different doses of minocycline (*p*-value shown on graph) and varying levels of AT-2 HIV or influenza on levels of TRAIL, IFNα, and IFNβ.

We next investigated whether minocycline could inhibit type I IFN responses in PBMCs by treating healthy human PBMCs with 0, 20, or 40 μM minocycline and increasing amounts of AT-2 HIV. Cells were analyzed after 18 hours for TRAIL surface expression by flow cytometry, and levels of IFNα and IFNβ in the supernatants were measured by ELISA. AT-2 HIV stimulated the production and secretion of IFNα (Fig. 2A; *p*<0.0001) and IFNβ (Fig. 2B; *p*<0.0001) by PBMCs in a dose-dependent manner, culminating in upregulation of TRAIL surface expression on lymphocytes (Fig. 2C; *p*<0.0001) that peaked with 300 ng/mL AT-2 HIV. Minocycline treatment decreased the production of IFNα (Fig. 2A; *p*<0.0001) and IFNβ (Fig. 2B; *p*<0.0001) and attenuated TRAIL upregulation in response to AT-2 HIV (Fig. 2C; *p* = 0.006).

To determine whether this effect was HIV-specific, and given the similarities in cytokine responses between HIV-1 and influenza, we stimulated PBMCs with varying doses of influenza virus with or without minocycline treatment. Influenza, like AT-2 HIV, induced robust IFNα (Fig. 2D; *p*<0.0001), IFNβ (Fig. 2E; *p*<0.0001), and TRAIL production (Fig. 2F; *p*<0.0001) in the PBMCs. Minocycline treatment tempered this response, yielding significantly lower levels of IFNα (Fig. 2D; *p* = 0.0004) and IFNβ (Fig. 2E; *p* = 0.0003), which suggests that minocycline has a broad inhibitory effect on anti-viral IFN signaling in PBMCs. Minocycline also prevented influenza-mediated TRAIL induction on lymphocytes in the PBMCs in a dose-dependent manner (Fig. 2F; *p* = 0.0003), again suggesting that this effect is not specific to HIV.

Representative flow cytometry gating of TRAIL expression on lymphocytes in a PBMC culture is shown in Fig. 2G for AT2-HIV stimulation and Fig. 2H for influenza (FLU) stimulation. In both cases, lymphocytes were gated on by FSC and SSC morphology. Flow cytometry scatter plots are representative of 4 experiments with AT-2 HIV and 3 experiments with influenza.

### Minocycline Prevented Upregulation of CTLA-4 and IDO in CD4+ T Cells and pDCs

**Figure 3:**
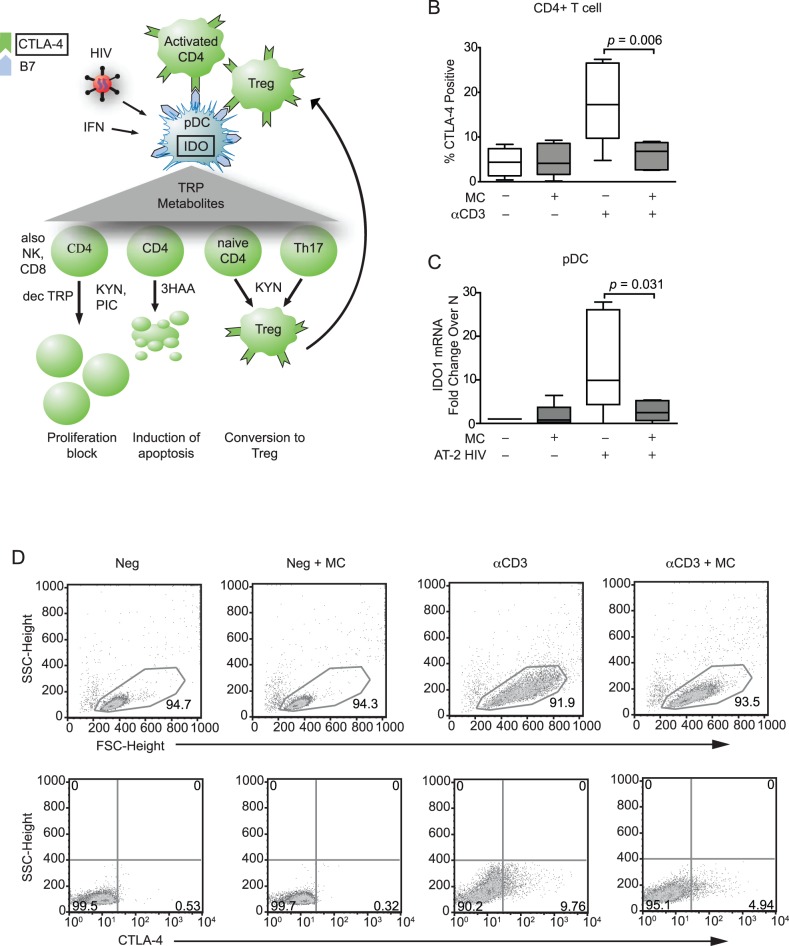
Minocycline prevents CTLA-4 and IDO expression in CD4+ T cells and pDCs. (**A**) IDO can be induced in pDCs by engagement of B7 receptors with CTLA-4 on CD4+ T cells or by stimulation with IFNα, β, γ, TNFα, TGFβ, or HIV. IDO converts the amino acid tryptophan (TRP) into L-formylkynurenine, initiating the production of a cascade of TRP metabolites that block T cell proliferation, induce T cell apoptosis, and convert CD4+ cells into Tregs. KYN: kynurenine; PIC: picolinic acid; TRP: tryptophan; 3HAA: 3-hydroxy anthranilic acid. (**B**) CD4+ T cells were isolated from blood of healthy human donors and activated with anti-CD3 with or without 20 μM minocycline (*n* = 6 different donors). After 24 hours minocycline was replenished and some wells were stimulated with IFNα and IFNβ. After 48 hours total cells were analyzed by flow cytometry for CTLA-4. (**C**) pDCs were isolated from blood of healthy human donors and exposed to AT-2 HIV with or without 20 μM minocycline (*n* = 6 different donors). After 18 hours RNA was harvested for IDO1 qRT-PCR. CTLA-4 data were analyzed by paired *t*-test. IDO mRNA was analyzed by Wilcoxon signed-rank test. (**D**) Representative flow cytometry gating of CTLA-4 expression on isolated CD4+ T cells.

As diagrammed in Fig. 3A, in the second arm of HIV/SIV pathogenesis CTLA-4 and IDO work in tandem to increase the number of suppressive DCs and Tregs in lymphoid tissues, further compromising immune responses already damaged by chronic ISGs [42]. Because CTLA-4-expressing T cells can induce IDO in pDCs, we examined whether minocycline could modulate CTLA-4 expression *in vitro* by activating CD4+ T cells with anti-CD3 in the presence or absence of minocycline (Fig. 3B). Minocycline maintained CTLA-4 at very low levels after exposure to anti-CD3 compared to anti-CD3 stimulation alone (*p* = 0.006). These data are in agreement with previous studies that demonstrated that minocycline attenuates CTLA-4 expression [86] and other markers of CD4+ T cell activation [53], [54]. However, IDO can also be induced in pDCs directly by virus or by cytokines such as type I IFN [34], [87]. Therefore, we stimulated pDCs with AT-2 HIV in the presence or absence of minocycline and analyzed IDO1 mRNA expression. pDCs that were treated with virus and minocycline expressed significantly less IDO1 mRNA than with virus alone (*p* = 0.031). IDO2, a variant of IDO1 that also catabolizes tryptophan, was not detected in any of the samples (data not shown).

Representative flow cytometry gating of CTLA-4 expression on isolated CD4+ T cells for all four treatment conditions is shown in Fig. 3D. Cells were gated on by FSC and SSC morphology.

### Minocycline Downregulated Activation-induced Genes Fas, CD25, and Caspase-3 but not IFN-stimulated Genes in Spleens of Chronically SIV-infected Pigtailed Macaques

To determine whether our *in vitro* findings could be recapitulated *in vivo*, we examined whether minocycline had an effect on IFN, IDO, and apoptosis-inducing factors TRAIL, FasL, and Fas in archived spleen samples from SIV-infected pigtailed macaques [62]. RNA was harvested from spleens from uninfected macaques, SIV-infected macaques in late stage infection, or SIV-infected macaques treated with either minocycline or cART and analyzed for viral RNA and various markers of immune activation.

**Figure 4:**
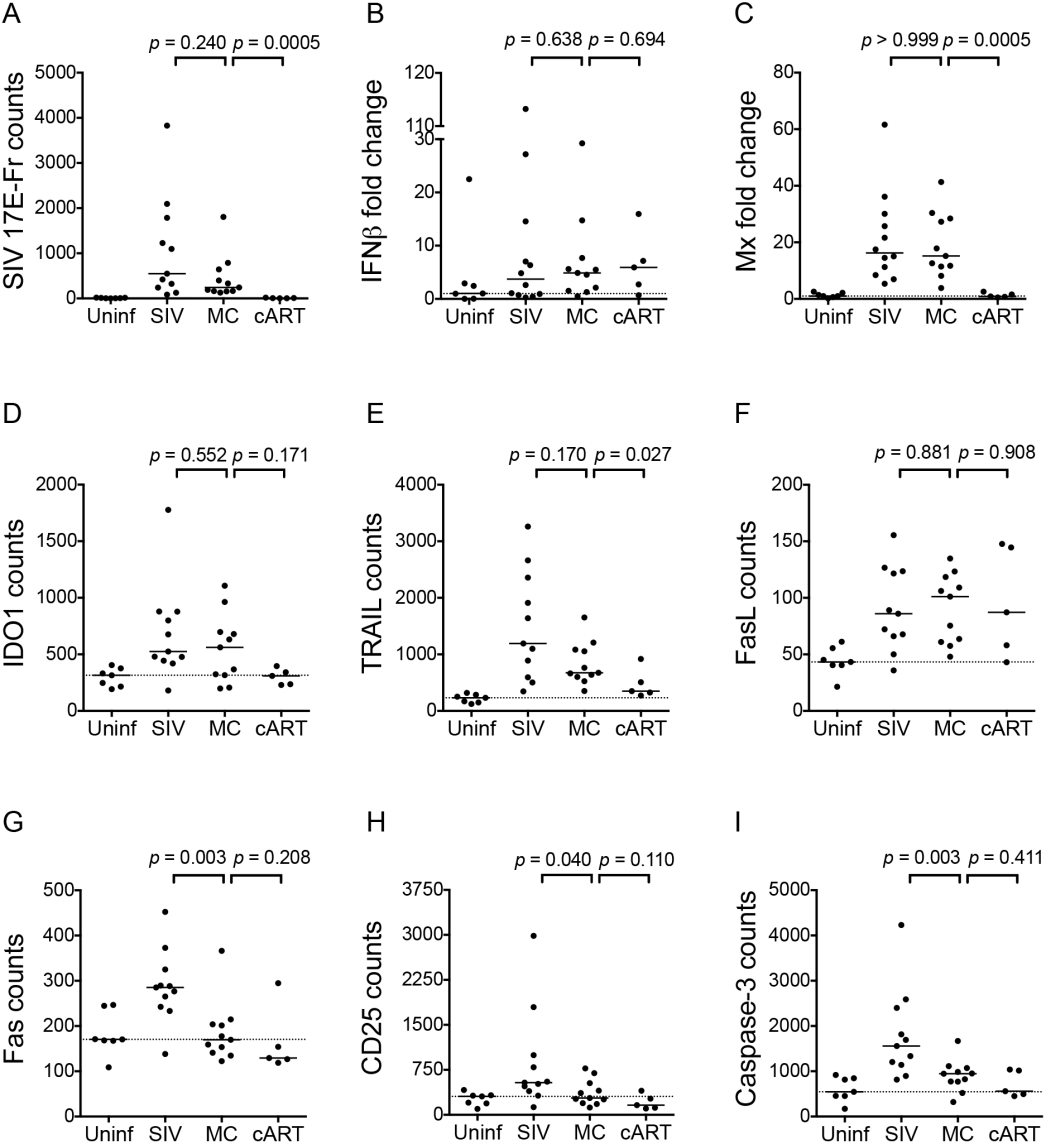
Minocycline attenuates activation but not IFN responses in spleens of SIV-infected pigtailed macaques. (**A–I**) The RNA expression of virus and 8 genes putatively involved in HIV/SIV pathogenesis were analyzed in RNA from archived spleens of uninfected controls (Uninf; *n* = 7), chronically SIV-infected pigtailed macaques (SIV; *n* = 11), chronically SIV-infected pigtailed macaques treated with minocycline starting at day 21 p.i. (MC; *n* = 11), and SIV-infected pigtailed macaques treated with cART starting at day 12 p.i. (cART; *n* = 5). All genes were analyzed by Nanostring nCounter analysis with the exception of IFNβ (**B**) and Mx (**C**), which were analyzed by qRT-PCR. Nanostring data are represented as counts; qRT-PCR data are represented as fold change over the median of 7 uninfected animals, although statistics were performed on delta Ct values prior to transformation. Solid lines denote medians; dashed lines represent the medians of uninfected controls. Data were analyzed by Mann-Whitney test.

We first measured the viral loads in the spleen because minocycline has previously been shown to reduce viral replication both *in vitro* and *in vivo* [51], [52] and can also inhibit HIV reactivation from latency [88]. Minocycline reduced median viral loads in the spleens of SIV-infected animals by 50%, but this decrease was not significant (Fig. 4A; *p* = 0.240). Despite high levels of virus in the chronically SIV-infected animals, IFNβ expression in spleen was not significantly elevated above uninfected controls during chronic infection (Fig. 4B; *p* = 0.157, Uninf vs. SIV). This may have been due to the wide spread in IFNβ expression levels, with one control animal in particular having very high levels. Though IFNβ was not significantly increased during chronic infection, we also measured the ISG Mx as a surrogate marker of total type I IFN responses because even small amounts of type I IFN can induce robust downstream responses. Mx was significantly elevated in chronically SIV infected animals compared to uninfected controls (Fig. 4B; *p*<0.0001). Minocycline treatment had no effect on IFNβ (Fig. 4B; *p* = 0.638) or Mx (Fig. 4C; p>0.999) transcriptional expression compared to SIV alone. Similarly, minocycline did not significantly reduce expression of IDO1 (Fig. 4D; *p* = 0.552) or the death ligands TRAIL (Fig. 4E; *p* = 0.170) and FasL (Fig. 4F; *p* = 0.881) *in vivo.*

In contrast to the lack of effect on the death ligands TRAIL and FasL, minocycline potently downregulated expression of the death receptor Fas (Fig. 4G; *p* = 0.003). Minocycline also significantly downregulated expression of the activation marker CD25 (Fig. 4H; *p* = 0.040) in the SIV-infected spleens. Finally, we examined expression of caspase-3, an important molecule in both extrinsic and intrinsic apoptosis signaling pathways, because several studies in a variety of disease models have demonstrated that minocycline alters caspase-3 transcription and activation [46], [89]–[91]. In our SIV-infected macaques minocycline potently reduced caspase-3 mRNA levels (Fig. 4I; *p* = 0.003).

### Comparison of Minocycline with Combination Antiretroviral Therapy (cART)

Given the significant effects of minocycline on various markers of activation-induced cell death in the spleens of SIV-infected macaques, we next determined how minocycline treatment compared to cART, the standard of care for HIV-infected individuals. cART treatment reduced the median expression levels of all genes back to uninfected levels, with the exception of IFNβ and FasL which were unchanged with either minocycline or cART treatment compared to spleens from SIV infected animals. As expected, cART treatment was significantly more effective than minocycline at reducing SIV viral load and the ISGs Mx and TRAIL in the macaque spleens (Fig. 4; MC vs. cART, *p* = 0.0005 for SIV, *p* = 0.0005 for Mx, *p* = 0.027 for TRAIL). Although cART medians were consistently lower than minocycline’s across all inflammatory genes, cART was not significantly more effective than minocycline at reducing expression of IDO1 (Fig. 4D; *p* = 0.171), FasL (Fig. 4F; *p* = 0.908), Fas (Fig. 4G; *p* = 0.208), CD25 (Fig. 4H; *p* = 0.110), or caspase-3 (Fig. 4I; *p* = 0.411).

## Discussion

Differences between NH and NNH suggest that chronic induction of the IFN and IDO pathways are at the root of immune pathogenesis seen in HIV infection [42]. In this study, minocycline had potent activity against IFN, IDO, and activation pathways in an acute model of HIV infection *in vitro,* which culminated in reductions in TRAIL expression on both pDCs and CD4+ T cells. We also observed reductions in IFN responses and TRAIL expression in minocycline-treated PBMCs exposed to either infectious influenza virus or AT-2 HIV. However, in spleens from chronically SIV-infected pigtailed macaques, minocycline did not affect IFNβ, Mx, IDO, TRAIL, or FasL but did significantly reduce activation-induced genes Fas, CD25, and caspase-3. Overall, these data suggest that minocycline attenuates markers of activation-induced cell death, a major component of HIV pathogenesis, but that testing for inhibition of type I IFN responses is more complex than can be discerned from our *in vitro* model.

In our *in vitro* model of acute infection, minocycline blocked both IFN- and activation-induced TRAIL, as seen by inhibition of AT-2 HIV-triggered IFNα and IFNβ responses in pDCs as well as prevention of TRAIL upregulation on CD4+ T cells activated with αCD3 antibody. The suppression of pDC IFN production could also be linked to modulation of pDC activation by minocycline; this will need to be confirmed in future studies. Importantly, minocycline also prevented TRAIL upregulation on CD4+ T cells stimulated with both αCD3 antibody and exogenous IFNα and IFNβ. These data showed that minocycline suppressed TRAIL even in a complex immune environment consisting of both TCR activation and IFN signals and led us to examine TRAIL in the chronically infected spleens.

Minocycline reduced TRAIL expression in the SIV-infected spleens by 50% but this change was not significant. This partial inhibition of TRAIL is consistent with our data that showed minocycline blocked only one of the two modes of TRAIL induction *in vivo*; i.e., minocycline reduced cellular activation (Fas and CD25) but did not reduce type I IFN responses (neither IFNβ nor the ISG Mx). Mechanistically, TRAIL upregulation on T cells through TCR activation/signaling is dependent on protein kinase C (PKC) translocation and Ca^2+^ mobilization [92]. Minocycline has previously been shown to inhibit these two mechanisms in other cell types and in isolated mitochondria [93]–[95], suggesting that the effects we observed on activation-induced TRAIL in T cells might be due to inhibition of PKC and/or Ca^2+^ mobilization by minocycline.

Despite not finding evidence for blockade of type I IFN signaling in the SIV-infected spleens, our experiments with influenza virus showed that minocycline’s inhibitory effects on type I IFN and TRAIL in *in vitro* PBMCs were not specific for inactivated viruses nor were they specific for HIV. These results are compelling because a cytokine storm is widely associated with influenza pathogenesis. Additionally, two groups recently reported that pDCs may participate in the cytokine storm observed in pathogenic influenza infections [16], [96], which posits a need for therapeutics that could modulate pDC cytokine responses in pathogenic influenza as well as HIV infection. Future studies must address whether minocycline’s inhibition of influenza-induced IFN responses *in vitro* can be recapitulated in an *in vivo* model or suffer from the same pitfalls as translation of HIV-induced IFN responses from *in vitro* to *in vivo* models.

In contrast to the lack of an effect on type I IFN responses in the SIV-infected spleens, minocycline treatment showed a trend towards reduced SIV RNA levels in spleen compared to infected, untreated animals, reducing viral loads by approximately 50%. Previous studies from our group and others have demonstrated that minocycline reduces viral replication in macaques and in a humanized mouse model of HIV infection, as well as *in vitro* in human and macaque primary macrophages and lymphocytes [51], [52], [86], [88]. However, in a recent pilot study of seven HIV-infected individuals not on anti-retroviral therapy, viral loads in both CSF and plasma were unchanged with an eight-week course of minocycline treatment [97]. Thus our findings of a trend towards reduction of viral loads in the spleens of SIV-infected animals may reflect a broader picture that is emerging of a more marginal effect of minocycline on viral loads during chronic infection, particularly in patients, than previously proposed.

Given the trend towards reduced splenic viral loads *in vivo* and minocycline’s robust inhibition of type I IFN responses in our *in vitro* model of acute infection, our finding that minocycline had no effect on IFNβ, Mx, or IDO transcript levels in the chronically infected spleen samples was surprising. IDO is induced by several other cytokines, including IFNγ and TFNα, but these have also been shown to be reduced by minocycline in *in vitro* studies [57], [58], [61], [98] as well as in a mouse model of vaginal mucosal inflammation [60]. However, in a pilot study in HIV-infected patients, minocycline did not affect plasma or CSF levels of neopterin, a marker of macrophage/microglial activation that is also induced by IFNγ [97]. It is possible that a higher dose of minocycline would be required to recapitulate *in vitro* findings for IFN and ISGs such as IDO *in vivo*.

Alternatively, it was recently reported that HIV-infected cells may serve as a more potent stimulator of type I IFN in PBMCs than cell-free virions [99]. It is conceivable that minocycline may not have as strong of an effect on IFN signaling mediated through contact with infected cells as opposed to free virus. In a pilot experiment with 3 donors using HIV-infected peripheral blood leukocytes co-cultured with autologous target PBMCs, minocycline inhibited TRAIL induction in pDCs but not in T cells as examined by flow cytometry (unpublished data). In contrast, minocycline reduced TRAIL induction in both pDCs and T cells from the same 3 donors after exposure to the TLR3 agonist poly I:C, suggesting that minocycline’s efficacy against type I IFN signaling may be dependent on both the cell type and source of immune stimuli.

Additionally, there may be differences in the mechanisms for IFN and ISG production during acute versus chronic SIV/HIV infection. For example, while pDCs are known to be major contributors to IFN responses in acute infection [81]–[83], their number and function in chronic infection are controversial, with some studies reporting higher numbers and/or higher IFN-producing capabilities of pDCs in chronically infected tissues [77]–[79], [82], [100], while others have reported depletion and/or dysfunction of pDCs in chronically infected tissues [80], [101]. Chronically infected animals also tend to have a smaller type I IFN fold induction compared to acutely infected animals [102]–[104], perhaps in part because of pDC dysfunction. In addition to different cellular sources of IFN, other variables potentially explaining the discordant findings on minocycline’s effectiveness against type I IFN responses *in vitro* versus *in vivo* include tissue selection, stage of disease, virus subtype, noncanonical IFN signaling [105] or alternative signaling pathways leading to ISG expression such as the TGFβ/IDO axis [35], [106].

We focused on TRAIL, a TNF family death ligand, as a representative downstream IFN effector molecule in this study because it is consistently elevated in SIV/HIV infection of NNH [9], [28], [107]–[111]. However, there is controversy over the expression of TRAIL’s death receptors, DR4 and DR5. Stary *et al.* and Kim *et al.* reported elevated DR4, but not DR5, by flow cytometry of circulating CD4+ T cells from HIV-infected patients [28] and SIV-infected rhesus macaques [111]. Herbeuval *et al.* demonstrated elevated DR5 mRNA expression in tonsillar lymphoid tissues from HIV progressors compared to nonprogressors [9]. In contrast, Chehimi *et al.* were unable to detect significant changes in DR5 by flow cytometry of CD4+ T cells from viremic individuals [13].

Because of these controversies surrounding DR4 and DR5 expression, we also examined expression of Fas and FasL in the SIV-infected spleens. Similar to TRAIL induction, Fas and FasL are highly regulated at the transcriptional level by either TCR activation or type I IFN signaling [92], [104], [112], [113], although *in vivo* data in mice suggest that FasL expression is more susceptible to IFN signaling than Fas [114]. We found that minocycline potently suppressed Fas (*p* = 0.003) but did not change FasL expression, consistent with our findings that minocycline reduced activation genes such as CD25 but not type I IFN *in vivo*. These data also support a greater role for IFN in the induction of death ligands than death receptors as others have found [114].

Finally, we also examined expression of caspase-3, a downstream mediator of both intrinsic and extrinsic apoptosis pathways that has been shown to be elevated in HIV/SIV infection [115], [116]. Minocycline’s protective effects against apoptosis in neurodegenerative diseases [45], [46] and fulminant hepatitis [89] have recently been attributed, in part, to reductions in caspase-3 expression and activation [46], [89]–[91]. We reproduced those findings in SIV-infected pigtailed macaques, demonstrating that minocycline-treated animals had significantly lower caspase-3 expression than infected, untreated animals (*p* = 0.003). By inhibiting caspase-3 expression, minocycline may attenuate not only TRAIL-mediated apoptosis but also other mechanisms of cell death. However, caspase-3 is also regulated at the protein level, and minocycline treatment did not completely reduce caspase-3 expression to uninfected control levels, so some degree of apoptosis could continue.

In comparison to minocycline, cART-treated animals consistently had lower expression of inflammatory and apoptotic genes, although only SIV/17E-Fr, Mx, and TRAIL mRNA levels were significantly different between the two treatments. The difference between splenic viral loads is not surprising (*p* = 0.0005, MC vs cART), because cART inhibits virus replication directly, whereas minocycline mediates its anti-viral effects indirectly by reducing cellular activation and thereby reducing the susceptibility of cells to infection. However, minocycline did reduce Fas, CD25, and caspase-3 to levels that were not significantly different from those of cART-treated animals. The overall modulation of the genes in this study, combined with other published mechanisms in HIV/SIV models [51], [52], [55], [86], [88], [117]–[119], suggests that immunomodulatory drugs such as minocycline might provide an important adjunct to cART [88].

However, despite the promising results obtained with minocycline in several animal models of inflammatory disorders, pilot studies in HIV-infected individuals have yet to yield significant changes in inflammatory markers such as neopterin, CCL2, or cognitive impairment [97], [120], [121]. Longer trials and perhaps earlier dosing with minocycline may be necessary, as the importance of early versus late initiation of minocycline treatment has been previously demonstrated [117]. In the present study, minocycline treatment was initiated at 21 days p.i., an asymptomatic timepoint occurring after the acute phase of infection but before the establishment of chronic infection and emergence of clinical symptoms. This is in contrast to the recent clinical trials, in which treatment was given to patients already displaying evidence of advanced, chronic infection (indicated by low CD4 counts, time since HIV diagnosis, and/or presence of neurocognitive impairment) [97], [120], [121]. Thus, particular attention should be paid towards whether minocycline can *prevent* immune pathogenesis as compared to reversing it. Future studies should also determine which of the myriad properties of minocycline are critical for its beneficial effects on HIV/SIV pathogenesis in animal models; these findings could then assist in discovery of drugs with enhanced pharmacological properties to improve efficacy in human trials.

The need for an adjunct for cART stems from evidence that, despite robust suppression of viral replication, small elevations in genes such as TRAIL [13], [108], [122], DR4 [28], [122], DR5 [108], Fas [108], FasL [108], caspase-3 [115], and IDO activity [15] persist in patients on cART, although these differences are not always significant and vary between cell and tissue types examined. Remarkably, in this study we found that cART treatment restored inflammatory gene expression back to uninfected control levels in spleen, with the exception of IFNβ (which was not significantly different between uninfected animals and chronically infected animals) and FasL. Additionally, despite viral suppression, one of the five cART treated animals consistently had higher gene activation than the rest of his group and may have been a candidate for adjunct therapy. The lingering immune activation seen in this animal may be analogous to the residual immune activation seen in some patients on cART, activation that is thought to contribute to comorbidities such as HIV-associated neurological disease. We propose the further study of immunomodulatory agents as adjuncts to cART, with the goal of reducing chronic immune activation back to uninfected control levels and improving the quality of life of HIV-infected individuals. Finally, our results with influenza, in conjunction with a recent report on the protective anti-inflammatory effects of doxycycline during influenza-associated pneumonia in mice [123], suggest that the tetracycline class of compounds may be useful in other viral diseases as well in which robust inflammatory cytokine responses and immune hyperactivation are associated with pathogenesis.

## Supporting Information

Figure S1 ***In vivo*** **spleen data normalized to RPS9 housekeeping gene.** In an alternative analysis to the one shown in Figure 4, spleen Nanostring nCounter data were normalized to the geometric mean of positive controls and then against the ribosomal gene RPS9, which was the traditional housekeeping gene that showed the least variance in expression between groups of animals.
(TIFF)

Table S1 **Raw Nanostring nCounter data.** RNA from spleens of SIV-infected pigtailed macaques were analyzed for expression of genes putatively involved in HIV/SIV pathogenesis. The genes listed were part of a larger CodeSet of 116 genes. Because of the high variability in expression of the 11 housekeeping genes despite equal amounts of RNA being loaded, the 3 genes that we found to be most stably expressed in our Nanostring nCounter CodeSet (CD2, CD5, and CD7) were also included because they were used for normalization purposes.
(TIFF)

Checklist S1 **ARRIVE guidelines checklist.** Information on the study design of the animal studies is reported in this checklist.
(DOC)
