# Supplementary figures and images for: Attenuation of Pathogenic Immune Responses during Infection with Human and Simian Immunodeficiency Virus (HIV/SIV) by the Tetracycline Derivative Minocycline
Source: PLoS One. 2014 Apr 14;9(4):e94375. doi: 10.1371/journal.pone.0094375 (PMC3986096; doi:10.1371/journal.pone.0094375)

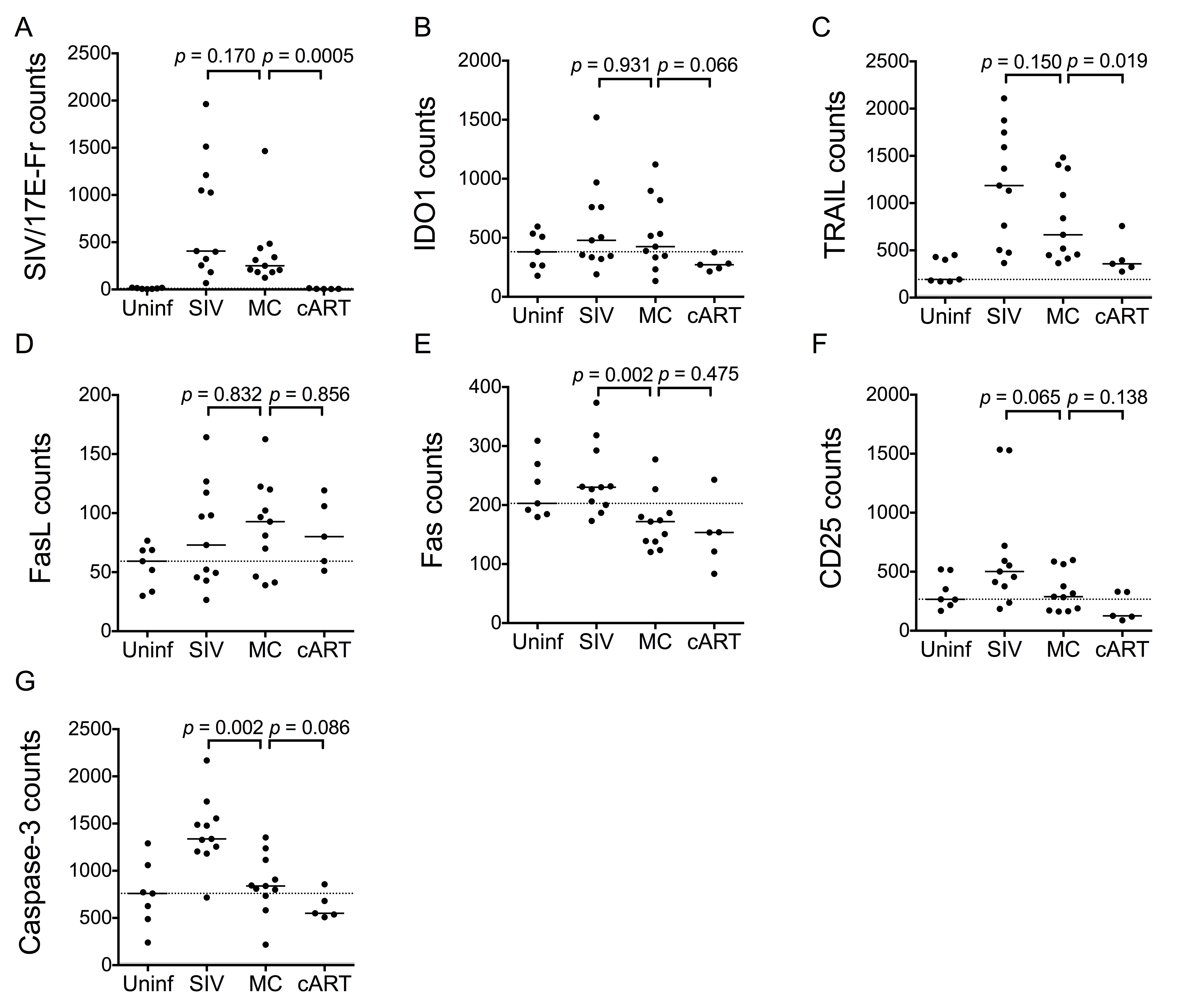

Supplement: Figure S1 — In vivo spleen data normalized to RPS9 housekeeping gene. In an alternative analysis to the one shown in Figure 4, spleen Nanostring nCounter data were normalized to the geometric mean of positive controls and then against the ribosomal gene RPS9, which was the traditional housekeeping gene that showed the least variance in expression between groups of animals. (TIFF) [file pone.0094375.s001.tiff]
